# Northern Ireland farm-level management factors for recurrent bovine tuberculosis herd breakdowns

**DOI:** 10.1017/S0950268822001479

**Published:** 2022-10-05

**Authors:** L. P. Doyle, E. A. Courcier, A. W. Gordon, M. J. H. O'Hagan, P. Johnston, E. McAleese, J. R. Buchanan, J. A. Stegeman, F. D. Menzies

**Affiliations:** 1 Veterinary Epidemiology Unit, Department of Agriculture, Environment and Rural Affairs, Dundonald House, Upper Newtownards Road, Belfast BT4 3SB, UK; 2 Statistical Services Branch, Agri-Food and Biosciences Institute, Newforge Lane, Belfast BT9 5PX, UK; 3 Department of Agriculture, Environment and Rural Affairs, Veterinary Service Animal Health Group, Ballykelly House, 111 Ballykelly Road, Ballykelly, Limavady BT49 9HP, UK; 4 Department of Farm Animal Health, Faculty of Veterinary Medicine, University of Utrecht, Yalelaan 7, Utrecht, The Netherlands

**Keywords:** Bovine tuberculosis, case control study, cattle, chronic breakdowns, epidemiology, *Mycobacterium bovis*

## Abstract

Bovine tuberculosis (bTB) is a chronic, infectious and zoonotic disease of domestic and wild animals caused mainly by *Mycobacterium bovis*. This study investigated farm management factors associated with recurrent bTB herd breakdowns (*n* = 2935) disclosed in the period 23 May 2016 to 21 May 2018 and is a follow-up to our 2020 paper which looked at long duration bTB herd breakdowns. A case control study design was used to construct an explanatory set of farm-level management factors associated with recurrent bTB herd breakdowns. In Northern Ireland, a Department of Agriculture Environment and Rural Affairs (DAERA) Veterinarian investigates bTB herd breakdowns using standardised guidelines to allocate a disease source. In this study, source was strongly linked to carryover of infection, suggesting that the diagnostic tests had failed to clear herd infection during the breakdown period. Other results from this study associated with recurrent bTB herd breakdowns were herd size and type (dairy herds 43% of cases), with both these variables intrinsically linked. Other associated risk factors were time of application of slurry, badger access to silage clamps, badger setts in the locality, cattle grazing silage fields immediately post-harvest, number of parcels of land the farmer associated with bTB, number of land parcels used for grazing and region of the country.

## Introduction

Bovine tuberculosis (bTB) caused by *Mycobacterium bovis* is a zoonotic disease primarily affecting animals. Although cattle are the main hosts, the disease has been reported in many other farmed and wild animals [1]. A key element of the bTB eradication strategy for Northern Ireland [2] published in 2016 was the recognition that herds chronically infected with bTB (‘chronic herds’) should be investigated as a distinct entity for action and a package of measures developed so as to minimise their impact on bTB eradication. This present work which focuses on herds with recurrent breakdowns (recurrently disclosing bTB) complements previous Northern Ireland work on the subject [3, 4] and aims to provide an accumulating evidence base for the development of effective policies. Addition of this present work to the other studies [3, 4] on the subject of chronic bTB will bring alignment between Northern Ireland and study of such management factors already completed in Great Britain (GB) and the Republic of Ireland (ROI) [5–8].

Doyle *et al*. (2016) used data from a national database APHIS (Animal and Public Health Information System) to inform definitions for prolonged and recurrent bTB breakdowns in chronic herds [3, 9]. The follow-up study on management factors for prolonged bTB breakdowns found that purchase of infected animal(s) had the strongest association as the most likely source of infection for long duration bTB herd breakdowns followed by badgers and then cattle-to-cattle contiguous herd spread [4]. It also demonstrated that two subpopulations of prolonged bTB breakdowns exist in Northern Ireland, the first being beef fattening herds with main source continuous purchase of infected animals and a second group of primary production herds (dairy, beef cows and mixed) with risk from multiple sources [4]. This present work focuses on farm-level management factors for chronic bTB herds that had recurrent bTB herd breakdowns. In this context chronic recurrent infection is the situation where a herd with confirmed bTB, defined as officially tuberculosis withdrawn (OTW), has this status removed post-completion of statutory testing (derestriction) and replaced by officially tuberculosis free (OTF) status (allowing it to trade with other herds); with this OTF/OTW/OTF cycle repeating many times in some herds before infection is cleared. When a herd breakdown recurs as a result of disease disclosure at a post-outbreak herd test or as a result of a lesion at routine slaughter (LRS) before the post-outbreak herd test it is likely this occurred due to the presence of infected but undetected animals at the time of derestriction [9].

Breakdown recurrence in Northern Ireland at the 6 monthly and yearly post-outbreak herd tests measured as a percentage of the tests carried out in the previous year was 11% and 9%, respectively, which mimics that for the herd tests carried out at derestriction (allowing a herd to regain its OTF status), at 11% (DAERA unpublished data); its percentage only being surpassed by herd tests carried out at the beginning and during bTB herd breakdown events. Disclosures of bTB infection at the 6-month post-outbreak herd tests are demoralising for farmers and veterinarians, which are interpreted as a previously wasted effort and are viewed as simply returning the farm business to the beginning of a protracted disease control process. This also crucially erodes confidence in the integrity of the whole bTB eradication programme. In Ireland, bTB recurrence occurs when there are further bTB restrictions in the same herd within a specific time-frame resulting from either residual undetected infection in cattle, residual infection due to contamination of fomites (such as slurry), contiguous inter-herd spread or reinfection from local wildlife, however of the sources listed residual infection is thought to lead to recurrence more quickly [9]. Other studies on bTB recurrence in herds generally investigated some or all of these sources of infection [9], the imperfect sensitivity of the tuberculin test [10] or bTB herd breakdown risk factors such as number of bTB reactors, herd size, retention of inconclusive bTB reactors and the herd bTB history [8, 11–16]. The percentage of herds retaining bTB infected animals after herd derestriction has also been modelled (up to 21% in the high risk area in England) [17]. Our study is unique in that it looked for associations in recurrent bTB breakdowns to farm management factors for Northern Ireland herds, comparing situations where bTB breakdowns recur quickly (cases) to those which do not recur or recur at a slower rate (controls), thus adding to the body of knowledge on the subject.

### Study objective

The objective of this study was to use a case control study design to identify farm-level management factors associated with recurrent bTB herd breakdowns, using data collected during on-farm epidemiological investigations.

## Materials and methods

### Study design and data collection

**Fig. 1.**
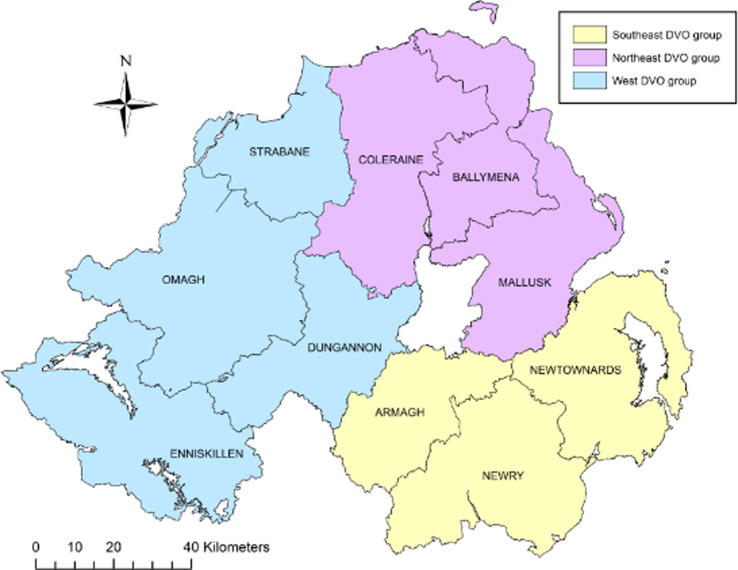
Northern Ireland DVOs aggregated into three groups: southeast, northeast and west.

A case control study was conducted on a study population consisting of all bTB herd breakdown investigations during the period 23 May 2016 to 21 May 2018. The methodology and model framework applied to this study were as previously applied [4, 18] but are summarised here for clarity. Data collection involved completion of an on-farm investigation form when one or more single intradermal comparative cervical test (SICCT) reactors or one or more confirmed LRS were disclosed in any Northern Ireland cattle herd. Confirmation of bTB in an LRS was defined as a positive histological and/or bacteriological culture result following laboratory examination. Investigations were carried out by trained Animal Health and Welfare Inspectors who visited each of the bTB breakdown farms. At each farm, an on-site questionnaire was completed (Supplementary Table S2) through face-to-face interview of the farmer, including identification of all herds contiguous to the bTB herd breakdown. Based on the completed questionnaire and local knowledge of the area, the DAERA Vet responsible for the bTB herd breakdown, where possible, determined the most likely source of infection for the breakdown. Questionnaire information along with data extracted from APHIS (herd size and location) were collated into Microsoft Access™ (Microsoft Corporation, Redmond, WA, USA). For our study, the 10 Divisional Veterinary Offices (DVOs) in Northern Ireland were aggregated into three groups according to their geographic location: southeast group (Armagh, Newry, Newtownards), west group (Dungannon, Enniskillen, Strabane, Omagh) and northeast group (Ballymena, Coleraine, Mallusk) (Fig. 1).

Cases were bTB herd breakdowns of less than a year in duration followed by at least two further bTB herd breakdowns within the following 2 years [3]. A disease investigation carried out on a bTB herd breakdown in the period 23 May 2016 to 21 May 2018 and which was preceded by at least two bTB herd breakdowns in previous 2 years was defined as a case [3]. These cases defined in this study, had an associated duration of <365 days to ensure mutual exclusivity to the cases used in the previous long duration study [4]. Controls were bTB herd breakdowns of less than a year in duration initiating during the study period (23 May 2016 to 21 May 2018) and which were linked to a maximum of one breakdown within the previous 2 years.

### Data analysis

Microsoft Access™ (Microsoft Corporation, Redmond, WA, USA) and R Version 3.4.0^1^ were used for data manipulations and R Version 3.4.0^1^ and Stata/SE 15^2^ were used for data analysis. The model framework used was binary logistic regression using purposeful selection of covariates [18] with the case definition forming the response variable. A total of 78 explanatory variables were derived from the on-farm questionnaire (see Supplementary Table S2) along with their associated factor levels. Initially all variables were tabulated using the duration case definition against each variable's factor levels. As variables were added or removed from the model, the Akaike information criterion (AIC) difference was calculated between the old and new proposed models, in order to determine if the proposal reduced AIC by a value greater than two [18]. Where the models were subsets of each other, the likelihood ratio test (LRT) was also used in order to determine if addition or removal of variables was significant at the *P* ≤ 0.05 level.

Initial analysis was by univariable logistic regression. Any variables containing low numbers (<10) of cases at any factor level, which could not be logically merged with another factor level were removed after univariable analysis. Remaining variables with *P* ≤ 0.25 were then analysed using a multivariable logistic regression model. The resultant model was further refined to produce a reduced multivariable logistic regression model, which utilised variables with *P* ≤ 0.05 from the first multivariable model. Following the fit of the reduced multivariable model, its estimated coefficients were compared to those in the initial multivariable model to determine if there was a magnitude change of >20%. This magnitude change known as 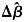
% indicated that one or more of the excluded variables were important in the sense of providing a needed adjustment effect of the variables that remained in the model [18]. Variables which formed the first multivariable model but were not included in the initial reduced multivariable model were added back individually; being retained if they contributed to the overall model and reduced 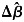
% to below 20%.

Furthermore, variables with *P* > 0.25 in the initial univariable analysis were also individually added back in, to determine if they contributed to the multivariable model thus producing the preliminary main effects model. The only continuous variable included in the preliminary main effects model was herd size. Fractional polynomial analysis [19] was applied to herd size in order to determine if it required scale transformation so as to satisfy the assumption of linearity in the logit outcome. Completion of this stage produced the main effects model. Using the variables present in the main effects model, all combinations of two-way interactions were statistically assessed using the LRT (*P*  ≤ 0.05); however, only those with probable biological significance were accepted as potential candidates for the model. Interaction terms accepted into the final model had an odds ratio (OR) calculated as a linear combination with their associated main effects (*β*_0_ + *β*_1_ + *β*_2_ + *β*_1_*β*_2_). The finalised model was then subjected to the Hosmer–Lemeshow goodness-of-fit test (decile sub-grouped) to determine its fit to the data. Table 1 details how the methodology was applied in this study to achieve the final multivariable model.

**Table 1.** Methods applied and results observed at each stage of the study model building process

Stage	Study methods	Study results
1	Univariable logistic regression applied to the 78 variables initially derived from farm questionnaire.	OR and associated *P*-value calculated for each level of all 78 variables.
2	Each of the 78 variables derived from the questionnaire was tabulated at each of its factor levels to determine number of cases/controls present at that factor level.	14 variables were removed where factor levels contained <10 cases and could not be logically merged with another level, leaving 64 variables to be carried forward for multivariable analysis.
3	Multivariable logistic regression applied to all variables with *P* ≤ 0.25 from stage 1 and not removed at stage 2.	Multivariable model containing 38 variables selected from stage 1 at the *P* ≤ 0.25 level and not removed at stage 2 (26 of the 64 variables had *P* > 0.25 and not reintroduced until stage 8).
4	Reduced multivariable model generated from variables with *P* ≤ 0.05 from stage 3.	Reduced multivariable model containing 10 variables selected from stage 3 at the *P* ≤ 0.05 level (28 of the 38 variables had *P* > 0.05).
5	Calculation of 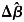 % (<20%) for reduced model produced at stage 4.	The variables ‘farm enterprise’ and ‘boundary fence upgrading’ had 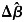 % >20%, thus some of the variables removed at stage 4 should be reassigned to model.
6	Individual reassignment of variables removed at stage 4 to the reduced model to determine if these variables contributed to the overall model.	Each of the 28 variables removed at stage 4 added back individually to determine if they contribute to overall model (LRT at *P* ≤ 0.05).
7	Reduced multivariable model refined by variable addition/removal to obtain 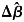 % <20% for all variables included in the multivariable model.	Refining of reduced multivariable model from stage 4 results in addition of three new variables: (1) silage fields after grazed with cattle, (2) herd size, (3) total parcels of land grazed. All 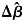 % for variables in the multivariable model at this stage of model building were <20%.
8	Individual reassignment of variables removed at stage 3 to the model produced at stage 7 to determine if these variables contributed to the overall model. Output from this stage formed the preliminary main effects model.	Each of the 26 variables removed at stage 3 were added back individually to determine if they contribute to the overall model (LRT at *P* ≤ 0.05). None of these variables were deemed to meet the criteria for addition to the model.
9	Fractional polynomial analysis to assess linearity of any continuous variables to the outcome.	Analysis results in the variable transformation of ‘herd size’ to a power of 0.5. ‘Herd size’ was added in its transformed state to the preliminary model to form the main effects model and was referred to as ‘herd size transformed’.
10	All combinations of two-way interactions (LRT at *P* ≤ 0.05 and judged as biologically significant) from main effects model were added to main effects model. Interactions retained or removed based on *P*-value (*P* ≤ 0.05) within the model and as to whether they lead to model improvement (AIC and LRT cut-offs already described). These interaction variable pairs were then evaluated as a linear combination (*β*_0_ + *β*_1_ + *β*_2_ + *β*_1_*β*_2_) with the main effects to determine their overall OR and associated CI (Table 3).	Two interaction terms were added to the main effects model of 13 variables to form the final model. ORs for these interactions in linear combination with their main effects were calculated and added to Table 3. Observing a dead badger on the farm in the last 3 years × Herd size transformed (LRT: *P* = 0.006) Total parcels of land grazed × Herd size transformed (LRT: *P* = 0.01)
11	Application of the goodness of fit test (Hosmer–Lemeshow goodness-of-fit test) and variable correlation analysis to the final model.	Hosmer–Lemeshow goodness-of-fit test result: *P* = 0.5987. Provides evidence at *P* ≤ 0.05 level of adequate goodness of fit.

## Results

A total of 2935 bTB herd breakdown investigations (192 cases and 2743 controls) were completed during the 2-year study period (23 May 2016 to 21 May 2018; summary details in Supplementary Table S1). These 2935 investigations were carried out in a total of 2730 individual Northern Ireland cattle herds as some herds had more than one investigation during the study period due to repeat herd breakdowns. Cases included in this work had a median duration of 164.5 days (interquartile range (IQR): 137.25–213), disclosed median number of SICCT reactors of 3 (IQR: 1–5) and a median time to previous herd breakdown of 169 days (IQR: 135.5–212). Controls were confirmed bTB breakdowns where there was no breakdown in the previous 2 years (*n* = 1924) or one breakdown (*n* = 819). The results from each stage of the model building process from univariable analysis through to the final multivariable model are detailed in Table 1. As a result of carrying out fractional polynomial analysis [19] on herd size it was transformed to herd size to power 0.5 and was then referred to as ‘herd size transformed’ in the subsequent analysis.

The results from the final model (Table 2) demonstrated that type of farming enterprise was a significant variable in the final multivariable model, however the factor level ORs were not statistically different from each other at the *P*  ≤ 0.05 level. In terms of farm enterprise type dairy enterprises (reference category) formed 43% of cases. The potential for badgers to access silage clamps was found to be statistically significant (*P* = 0.013; OR 1.752; 95% confidence interval (CI) 1.137–2.775) while the application of slurry/manure to farmland mostly in the spring time as opposed to continuously over the grazing season was protective (*P* = 0.27; OR 0.672; 95% CI 0.475–0.961). The ORs of a case in relation to geographical/other factors (Table 2) were as follows: where particular parcels of ground were associated with TB in cattle was 1.676 (95% CI 1.152–2.411); where there was partial upgrading of boundary fences in the last 3 years was 1.804 (95% CI 1.154–2.883); where there was full upgrade of boundary fences in the last 3 years was 1.247 (95% CI 0.801–1.981); where there was presence of badger setts locally but not on the investigation farm was 1.368 (95% CI 0.979–1.905); where there was any woodland on the farm or within 1.6 km from the farm was 0.648 (95% CI 0.470–0.895); where a farm is located in DVO west group was 1.328 (95% CI 0.881–2.037) and with DVO northeast group was 1.675 (95% CI 1.066–2.662); with silage fields after grazed by cattle was 1.918 (95% CI 1.117–3.528).

**Table 2.** Results of final multivariable case control study containing categorical and continuous variables (note interaction terms are included in Table 3)

Study variable categorical	Exposure level	Case (*n* = 192)		Control (*n* = 2743)		OR	95% CI	*P*-Value
		*n*	%	*n*	%			
Type of farming enterprise	Dairying	83	10.85	682	89.15	–	–	–
	Beef cows	36	3.36	1035	96.64	0.638	0.390–1.034	0.071
	Beef fattening	39	6.14	596	93.86	1.194	0.716–1.972	0.492
	Beef cows and fattening	16	5.23	290	94.77	0.644	0.345–1.143	0.148
	Mixed enterprise	18	11.39	140	88.61	1.127	0.608–2.002	0.693
Could badgers potentially access silage clamps?	No	29	2.94	959	97.06	–	–	–
	Yes	163	8.37	1784	91.63	1.752	1.137–2.775	0.013
Is slurry/manure applied mostly in spring time rather than continuously over grazing season?	No	54	7.64	653	92.36	–	–	–
	Yes	138	6.19	2090	93.81	0.672	0.475–0.961	0.027
Do you have particular parcels of land that you associate with TB in your cattle?	No	139	5.72	2293	94.28	–	–	–
	Yes	53	10.54	450	89.46	1.676	1.152–2.411	0.006
Any boundary fence with a neighbour upgraded in the past 3 years (where full upgrade is installation of a complete new fence)?	No	31	4.30	690	95.70	–	–	–
	Some upgrading	77	7.27	982	92.73	1.804	1.154–2.883	0.011
	Full upgrading	84	7.27	1071	92.73	1.247	0.801–1.981	0.338
Are you aware of any badger setts in the locality but not on your farm?	No	110	5.51	1887	94.49	–	–	–
	Yes	82	8.74	856	91.26	1.368	0.979–1.905	0.065
Have you seen a dead badger on your farm in the last 3 years?	No	162	5.86	2602	94.14	–	–	–
	Yes	30	17.54	141	82.46	9.477	2.989–29.863	0.000
Any woodland on the farm or within 1.6 km of the farm?	No	95	7.61	1154	92.39	–	–	–
	Yes	97	5.75	1589	94.25	0.648	0.470–0.895	0.008
DVO of the bTB breakdown	Armagh, Newry, Newtownards (southeast group)	37	4.47	790	95.53	–	–	–
	Dungannon, Enniskillen, Strabane, Omagh (west group)	88	6.26	1317	93.74	1.328	0.881–2.037	0.183
	Ballymena, Coleraine, Mallusk (northeast group)	67	9.53	636	90.47	1.675	1.066–2.662	0.027
Do you after graze your silage fields with cattle?	No or NA	15	3.94	366	96.06	–	–	–
	Yes	177	6.93	2377	93.07	1.918	1.117–3.528	0.026
bTB breakdown risk picked as most likely source by DAERA Vet	Source of infection not established (includes Other and Deer source)	44	4.88	857	95.12	–	–	–
	Cattle to cattle contiguous herd spread	39	5.86	627	94.14	0.890	0.554–1.424	0.629
	Purchase of infected animal(s)	33	6.35	487	93.65	1.349	0.797–2.266	0.260
	Carryover of previous infection	40	18.78	173	81.22	2.565	1.561–4.198	0.000
	Badgers	36	5.67	599	94.33	0.914	0.560–1.482	0.715
What is the total number of land parcels you use for grazing cattle?	One parcel of land used	33	4.09	774	95.91	–	–	–
	Two parcels of land used	45	6.12	690	93.88	0.806	0.253–2.543	0.714
	Three parcels of land used	57	9.06	572	90.94	1.45	0.476–4.422	0.512
	Four or more parcels of land used	57	7.46	707	92.54	3.423	1.178–10.096	0.024
Study variable	Exposure level	Mean (95% CI)				OR	95% CI	*P*-Value
		Case		Control				
Herd size transformed	Average herd size as number of animals over the time period 2016–2018	202.47 (181.54–223.40) (untransformed value)		130.39 (124.92–135.86) (untransformed value)		1.108	1.035–1.182	0.002

NA, not applicable.

With respect to source of infection for cases (Table 2), the OR for cattle-to-cattle contiguous herd spread was 0.890 (95% CI 0.554–1.424), for purchase of infected animal(s) source was 1.349 (95% CI 0.797–2.266), for carryover of infection source was 2.565 (95% CI 1.561–4.198) and for badger infection source was 0.914 (95% CI 0.560–1.482). The presence of a badger sett was recorded on 30% (95% CI 28.4–31.7) of investigations and of these 3% (95% CI 2.3–3.6) reported fencing the sett/latrine off. Of cases which were determined to have a breakdown source as carryover of infection 57.5% (95% CI 42.2–72.8) were dairy enterprises, 25% (95% CI 11.6–38.4) were beef cow enterprises, 15% (95% CI 3.9–26.1) were beef fattening enterprises and 2.5% (95% CI 0.0–7.3) were other mixed enterprises.

**Fig. 2.**
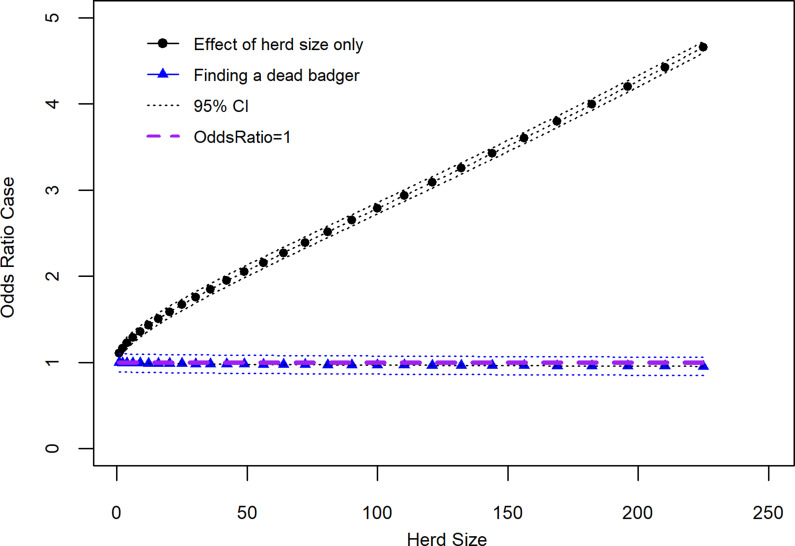
Recurrence case OR for observing a dead badger on a farm given effect of increasing herd size (variable herd size graphed in untransformed state).

**Fig. 3.**
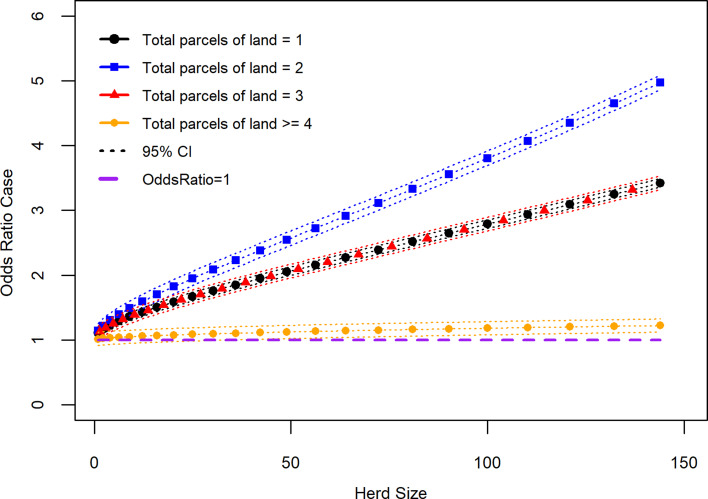
Recurrence case OR for bTB herd breakdown by number of parcels of land used for grazing given effect of increasing herd size (variable herd size graphed in untransformed state).

Along with the main effects, addition of two interaction terms, ‘finding a dead badger on your farm in the past three years’ × ‘herd size transformed’ and ‘total parcels of land grazed’ × ‘herd size transformed’ improved the model. A third interaction term, ‘finding a dead badger on your farm in the past three years’ × ‘slurry/manure application mostly in the spring rather than continuously over the grazing season’, was not included as it was deemed biologically implausible. The results of the linear combination of the two interaction terms included in the model and their associated main effects (*β*_0_ + *β*_1_ + *β*_2_ + *β*_1_*β*_2_) are shown in Table 3 and Figures 2 and 3. Results from Table 3 show that the OR of a case where a dead badger was observed on the farm in the previous 3 years and there was an increase in herd size transformed by one was 0.997 (95% CI 0.880–1.070). Figure 2 shows the effect on the recurrence case definition of increasing herd size (untransformed) in situations where a dead badger was observed on the farm in the previous 3 years. The results of Figure 2 show that when herd size is taken into account, the finding of a dead badger does not lead to an OR of a recurrent case which differs significantly from one.

**Table 3.** Results of final multivariable case control study containing calculated effects for the two-way interactions included in the model

Variable interactions		OR	95% CI
Observing a dead badger on your farm in the last 3 years?	Herd size transformed (increase in value by one)		
Yes	No	1	–
Yes	Yes	0.997	0.880–1.070
What is the total number of land parcels you use for grazing cattle?	Herd size transformed (increase in value by one)		
One parcel of land used	No	1	–
	Yes	1.108	1.035–1.182
Two parcels of land used	No	1	–
	Yes	1.143	1.023–1.277
Three parcels of land used	No	1	–
	Yes	1.108	0.997–1.232
Four or more parcels of land used	No	1	–
	Yes	1.017	0.919–1.126

Figure 2 also shows that there is a strong association between increased OR of a recurrent breakdown and increasing herd size. Within the recurrence case group, the mean herd size was 202 (95% CI 182–223) and median herd size 158 (IQR: 93–266) animals. In this study control herds were also bTB breakdowns with mean herd size 130 (95% CI 125–136) animals and median 80 (IQR: 38–172). In the Northern Ireland cattle industry as a whole average herd size in 2019 was 76.0 animals (2019 TB testing data) [20].

The interaction variable ‘total parcels of land grazed’ × ‘herd size transformed’ had a significant effect on addition to the main effects model (LRT: *P* = 0.01; Table 3). The OR of a case where one parcel of land is used for grazing cattle and there was an increase in herd size transformed by one had was 1.108 (95% CI 1.035–1.182), where two parcels of land was used and herd size transformed increased by one was 1.143 (95% CI 1.023–1.277), where three parcels of land were used and herd size transformed increased by one was 1.108 (95% CI 0.997–1.232) and with four or more parcels of land and herd size transformed increase by one was 1.017 (95% CI 0.919–1.126). Figure 3 shows the effect on the recurrence case definition of increasing herd size (untransformed) at the different levels of the number of land parcels used for grazing cattle variable. In terms of recurrent cases where one parcel of land was used to graze cattle this group was made up of 21.2% (95% CI 7.3–35.1) dairy enterprises, where two parcels of land was used to graze cattle 57.7% (95% CI 43.3–72.2) were dairy enterprises, where three parcels of land was used to graze cattle 49.1% (95% CI 36.1–62.1) were dairy enterprises where four or more parcels of land were used to graze cattle 38.6% (95% CI 25.9–51.2) were dairy enterprises.

## Discussion

This work delivers an insight into recurrent bTB breakdowns in Northern Ireland, complementing a previous study on chronic bTB which looked at prolonged breakdowns [4]. It looked specifically at farm management factors, an area which has not been previously investigated using Northern Ireland data and, by using cases disclosing three bTB breakdowns within 3 years, provides an unique comparison of the more extreme end of the spectrum for recurrence.

Previous work investigating prolonged bTB herd breakdowns [4] showed these to have a strongest association to purchase of infected animal(s) followed by badgers and then cattle to cattle contiguous spread. However, when herd type was considered, it was demonstrated that the main herd type contributing to the purchase of infection source was defined as beef fattening herds, while other primary production herds (dairy, beef cows and mixed) were at risk from multiple sources. In contrast, this present work, which considers recurrent bTB herd breakdowns, showed these to be strongly associated with carryover of infection most likely resulting from failure to clear herd infection during the breakdown period.

Data used in this study were derived from two sources: the farmer experiencing the bTB breakdown and a trained DAERA Veterinarians who managed the breakdown and allocated a source using standardised guidance. With our previous study [4] variables relating to purchase of cattle were identified by farmer and DAERA Veterinarians responses as significant contributors to prolonged breakdowns. This contrasts with this parallel study on recurrent breakdowns where variables relating cattle purchases were not significant management factors; a finding which is similar to that observed in GB [8]. An Irish study [9] hypothesised that persistent infection in a herd due to residual infection in cattle would be expected, on average to lead to recurrence more quickly than re-infection from other sources. Our results indicate that carryover of infection from a previous breakdown was the only level of this factor found statistically significant, hence concurring with the Irish study conclusions [9].

This result is not surprising given the imperfect sensitivity of the SICCT [10] and changes have been applied to the Northern Ireland bTB eradication programme aimed at increasing its effectiveness. Recent changes have included enhanced removal of inconclusive SICCT reactors [21–23], enhanced application of more stringent test cut-off values (severe interpretation) [21, 22], increased number of breakdowns subjected to a minimum 120 day restricted period and minimum two SICCT herd tests [21, 22], application of parallel interferon-gamma testing to enhance test sensitivity [20, 24] and also broader initiatives such as introduction in 2016 of a private veterinary practitioner contract, which aimed to improve overall quality of bTB testing [20].

In contrast to the DAERA Veterinarians who pointed to an unambiguous source of infection for recurrent breakdowns, farmers' responses returned associations to a much wider spectrum of management factors. Type of farming enterprise was a significant variable throughout the model building process. Dairy farming enterprise was taken as the baseline category and relative to it, none of the other levels showed a significant difference (*P* < 0.05). In terms of recurrent cases, 43.2% were dairy farm enterprises which is a large representation given that approximately 10% of Northern Ireland farming enterprises are defined as dairy [25], however results did not show dairy farm enterprises at a significantly greater risk than other herd types. Previous work in GB and ROI [8, 14] studied the effects of herd type in recurrent breakdowns, the former study including dairy herds as a risk factor, while the ROI study did not include it in the final multivariable model. However both studies [8, 14] acknowledged a relationship between herd type and herd size as influencing which of these variables appear in final models. Notably our study included both farm enterprise and herd size in the final model. As in GB and ROI, in Northern Ireland the relationship between herd type and farm enterprise shows that dairy farms are generally larger than beef breeding farms (average herd size 95 *vs.* 15 cows, respectively [25]). However, compared to the general population, our study only involved bTB breakdowns which are more likely to occur in larger herds (average control herd size was 130.4 (95% CI 124.92–135.86)). Herd size arguably provides a statement summarising the increased bTB risk exposures experienced relatively by large herds. These risks include the need to graze larger areas, risking increased contact with infectious wildlife, more purchase and movement of cattle, larger numbers of contiguous herds, more intensive management with higher production stress and, if infected, more difficulty in clearing infection due to the poor sensitivity of the SICCT allowing infected animals with false-negative results remaining in the herd [26, 27].

In addition to the direct risk from herd size there has been a move towards intensification in Britain and Ireland with a trend towards larger farms and decline in absolute numbers [26], probably making the problem more intractable (in Northern Ireland, there has been almost a 1% annual decline in the number of cattle herds over the last 15 years while cattle number have remained relatively constant [28]). With our study, herd size also had an interaction effect with both finding a dead badger on the farm in the previous 3 years and number of land parcels used for grazing cattle. In the case of finding a dead badger on the farm, this variable did not have a significant association to the case definition when interpreted at increasing levels of herd size (Fig. 2). A previous Northern Ireland study reported an association between the finding of badger carcases on the farm and bTB breakdown [29]. Indeed, even with number of parcels of land used for grazing cattle what appeared to be an increasing strength of association linked to more parcels became less clear when interpreted as an interaction with herd size (Fig. 3). When interpreted as an interaction with herd size, only the category using two parcels of land had a statistically significant OR greater than the baseline (one parcel); a group made up of 57.7% dairy enterprises. This result may reflect a situation in large herds where they are managed as several smaller units making it easier to clear infection from a particular group of cattle, which in terms of diagnostic test performance functions as a ‘small’ herd. The result for use of two parcels of land (odds of a recurrent case significantly greater than the baseline (one parcel)) is noteworthy given the high proportion of dairy herds in this group. This result could point to the situation that dairy herds are more likely to function as a single unit than other herd types, even when they operate on more than one parcel of ground. Further investigations are required to disentangle these findings especially its significance in relation to type of farm (dairy *vs.* non-dairy) [30].

As already noted [9] the recurrence of bTB breakdowns could originate from sources other than carryover of infection, such as contamination of fomites such as slurry, contiguous spread or reinfection from local wildlife. A significant reduction in the OR of a recurrent bTB breakdown was recorded when slurry was mostly applied in the spring time, rather than continuously over the grazing season. This factor may be indicative of farming intensity where land availability is limited to enable application of the majority of the slurry early in the grazing season. The direct risk of infection to cattle from the slurry application process [5] is probably increased if continued over the grazing season when direct exposure to cattle is more likely; however recent work showed the prevalence of *M. bovis* in faecal samples from bTB infected cattle was extremely low [31].

In terms of contiguous spread, recent upgrading of boundary fences was significant. However, the interpretation is unclear as it suggested that partial upgrade of boundary fences leads to an increase in recurrence while full upgrade was not a statistically significant factor level. One explanation might be that farmers suffering recurrent breakdowns were upgrading boundary fences as a reactive biosecurity measure, which was ineffective as, in many cases, the bTB source was carry-over of infection in their own herd. Another significant variable which could be related to both boundary fencing and wildlife in the locality was a farmer highlighting specific land parcels they associated with bTB in their cattle. This variable however has limitations in terms of its interpretation as such land parcels could be adjacent to herds at high risk of bTB breakdowns or favourable to wildlife/badger habitats, which if infected could act as a source for recurrent bTB herd breakdowns.

Two other significant variables related to badgers were access to silage clamps and the presence of badger setts in the locality but not on the investigation farm. However, similar to the previous study [4], results indicated that 30% of farms investigated claimed to have a badger sett present but only 3% of farms actively fence off setts to prevent cattle accessing them. Another study in Northern Ireland demonstrated that cattle visit badger setts/location three times more frequently than badgers visit cattle locations [32] affirming that this lack of such a biosecurity barrier may be an important finding in preventing this potential indirect infection pathway.

Grazing cattle on fields after silage removal was also significantly related to the cases. This variable could be a proxy for farms with more intensive production, also allowing more exposure to contiguous herds [33]. However, it is a practice which forces cattle to graze around a field perimeter possibly exposing them to badger setts and latrines which are more likely to be located in hedgerows in a Northern Ireland setting [34–37] and moreover, very unlikely to be fenced off.

As with our previous study [4], this study also showed that herds located in the DVO northeast group (Fig. 1) have the strongest statistical association to the recurrent bTB herd breakdowns. Also included and showing a reduced OR for its association to cases was the presence of woodland on the farm or within 1.6 km of the farm. Variables relating to woodland in the farm vicinity has been included in studies previously [5, 38] but were not significant. It is possible that in Northern Ireland woodland could act as a proxy for areas adjacent to coniferous plantations which are predominantly in areas of poorer land quality and smaller cattle herds.

In this study the final multivariable model contained 13 variables and given the use of a statistical cut-off level of *P* < 0.05 for variable selection, there is a possibility that at least one spurious variable has been included. It is also possible with a study design where DAERA Veterinarian's select a breakdown source of infection there is potential for a degree of subjectivity. However, the DAERA Veterinarian with their local knowledge, training and standardised guidance is best placed to make these assessments.

## Conclusions

One of the most important tasks a DAERA Veterinarian performs is investigating bTB herd breakdowns where, after careful consideration using standardised guidelines, they allocate a disease source. In the situation where there was a high-frequency bTB herd breakdown pattern (three individual bTB breakdowns within a 3-year period), this study showed that DAERA Veterinarians strongly associated the infection source to be carry-over of infection, indicating that the diagnostic tests have failed to clear herd infection in the time allocated to the restricted period for these cases. In terms of management factors associated with recurrent bTB herd breakdowns, the size of the herd and location in north-eastern Northern Ireland were significant, along with indicators of increased production intensity such as extended slurry spreading on grassland and after-grazing of silage fields. Herd type was a very strong contender variable in the final model, with dairy herds making up 43% of cases; however, factor level results did not show them to be statistically more at risk than other herd types. Even though badgers were not highlighted as a significant source of infection in this study, it cannot be ignored that so little effort is applied to create a biosecurity barrier between their setts/latrines and grazing cattle.

## Data Availability

The data that support the findings of this study are not publically available.

## Footnotes

1 R Core Team (2017). R: A language and environment for statistical computing. R Foundation for Statistical Computing, Vienna, Austria (https://www.R-project.org/).

2 StataCorp. 2017. Stata Statistical Software: Release 15. College Station, TX: StataCorp LP.

## Supplementary material

For supplementary material accompanying this paper visit http://doi.org/10.1017/S0950268822001479. click here to view supplementary material
