# Supplementary material for: Northern Ireland farm-level management factors for recurrent bovine tuberculosis herd breakdowns
Source: Epidemiol Infect. 2022 Oct 5;150:e176. doi: 10.1017/S0950268822001479 (PMC9987019; doi:10.1017/S0950268822001479)
Supplement: Supplementary file 1 [file S0950268822001479sup001.docx]

**Supplementary Material**

**Farm Level Management Factors for Recurrent Bovine Tuberculosis Herd Breakdowns**

L.P. DOYLE^1*^, E.A. COURCIER^1^, A.W. GORDON^2^, M.J.H. O’HAGAN^1^, P. JOHNSTON^3^, E. MCALEESE^3^, J.R. BUCHANAN^3^, J.A. STEGEMAN^4^, F.D. MENZIES^1^.

*^1^Veterinary Epidemiology Unit, Department of Agriculture, Environment and Rural Affairs, Dundonald House, Upper Newtownards Road, Belfast BT4 3SB, United Kingdom*

*^2^ Statistical Services Branch, Agri-Food and Biosciences Institute, Newforge Lane, Belfast, BT9 5PX, United Kingdom*

*^3^Department of Agriculture, Environment and Rural Affairs, Veterinary Service Animal Health Group, Ballykelly House, 111 Ballykelly Road, Ballykelly, Limavady, BT49 9HP*

*^4^ Department of Farm Animal Health, Faculty of Veterinary Medicine, University of Utrecht, Yalelaan 7, Utrecht, The Netherlands*

* Corresponding author. Address: Veterinary Epidemiology Unit, Department of Agriculture, Environment and Rural Affairs, Dundonald House, Upper Newtownards Road, Belfast BT4 3SB. Tel.: +44 2890765333. E-mail address: [liam.doyle@daera-ni.gov.uk](mailto:liam.doyle@daera-ni.gov.uk)

**Supplementary Table S1:** Tabulation of each of the 78 study variables (derived from the on-farm questionnaire) against the case definition.

| Study Variable | Description of Study Variable | Case | | Control | | Total | |
| --- | --- | --- | --- | --- | --- | --- | --- |
|  |  | n=192 | | n=2743 | | n=2935 | |
|  |  | n | % | n | % | n | % |
| FARMACT | Farming enterprise practised on farm |  |  |  |  |  |  |
|  | Milking cows | 83 | 10.85 | 682 | 89.15 | 765 | 26.06 |
|  | Beef cows | 36 | 3.36 | 1035 | 96.64 | 1071 | 36.49 |
|  | Beef fattening | 39 | 6.14 | 596 | 93.86 | 635 | 21.64 |
|  | Beef cows + Beef fattening | 16 | 5.23 | 290 | 94.77 | 306 | 10.43 |
|  | Other mixed enterprises | 18 | 11.39 | 140 | 88.61 | 158 | 5.38 |
|  |  |  |  |  |  |  |  |
| PEDIGREE | Registered pedigree animals present on farm |  |  |  |  |  |  |
|  | No | 118 | 5.94 | 1870 | 94.06 | 1988 | 67.73 |
|  | Yes | 74 | 7.81 | 873 | 92.19 | 947 | 32.27 |
|  |  |  |  |  |  |  |  |
| SIZECHNGE | Herd size change over past 10 years |  |  |  |  |  |  |
|  | Decrease | 18 | 5.33 | 320 | 94.67 | 338 | 11.52 |
|  | Same | 114 | 6.48 | 1644 | 93.52 | 1758 | 59.90 |
|  | Increased | 60 | 7.15 | 779 | 92.85 | 839 | 28.59 |
|  |  |  |  |  |  |  |  |
| WINTOUT | Any cattle wintered outside |  |  |  |  |  |  |
|  | No | 174 | 6.64 | 2448 | 93.36 | 2622 | 89.34 |
|  | Yes | 18 | 5.75 | 295 | 94.25 | 313 | 10.66 |
|  |  |  |  |  |  |  |  |
| SEPUNITS | Is part of the herd managed as a separate unit |  |  |  |  |  |  |
|  | No | 156 | 6.06 | 2420 | 93.94 | 2576 | 87.77 |
|  | Yes | 36 | 10.03 | 323 | 89.97 | 359 | 12.23 |
|  |  |  |  |  |  |  |  |
| TBSUBGRP | Has TB affected a particular sub group of the herd |  |  |  |  |  |  |
|  | No | 83 | 6.81 | 1136 | 93.19 | 1219 | 41.53 |
|  | Cattle <=30 months | 48 | 6.08 | 741 | 93.92 | 789 | 26.88 |
|  | Cattle >30 months | 61 | 6.58 | 866 | 93.42 | 927 | 31.58 |
|  |  |  |  |  |  |  |  |
| GRAZPAT | What grazing patterns are practised? |  |  |  |  |  |  |
|  | Not Applicable | 90 | 5.66 | 1499 | 94.34 | 1589 | 54.14 |
|  | Rotational | 40 | 5.24 | 723 | 94.76 | 763 | 26.00 |
|  | Set Stocking | 33 | 11.91 | 244 | 88.09 | 277 | 9.44 |
|  | Strip Grazing | 24 | 9.41 | 231 | 90.59 | 255 | 8.69 |
|  | Mixed | 5 | 9.80 | 46 | 90.20 | 51 | 1.74 |
|  |  |  |  |  |  |  |  |
| ZEROGRAZ | Are any cattle groups zero grazed |  |  |  |  |  |  |
|  | No | 179 | 6.33 | 2648 | 93.67 | 2827 | 96.32 |
|  | Yes | 13 | 12.04 | 95 | 87.96 | 108 | 3.68 |
|  |  |  |  |  |  |  |  |
| MIXGRAZ | Is there any mixed grazing of cattle and sheep |  |  |  |  |  |  |
|  | No | 153 | 6.61 | 2163 | 93.39 | 2316 | 78.91 |
|  | Yes | 39 | 6.30 | 580 | 93.70 | 619 | 21.09 |
|  |  |  |  |  |  |  |  |
| TRGHFEED | Is meal fed at unraised troughs or on the ground? |  |  |  |  |  |  |
|  | No | 167 | 6.23 | 2512 | 93.77 | 2679 | 91.28 |
|  | Yes | 25 | 9.77 | 231 | 90.23 | 256 | 8.72 |
|  |  |  |  |  |  |  |  |
| CLFCREEP | Are calf creep feeders used at pasture |  |  |  |  |  |  |
|  | No | 164 | 6.83 | 2238 | 93.17 | 2402 | 81.84 |
|  | Yes | 28 | 5.25 | 505 | 94.75 | 533 | 18.16 |
|  |  |  |  |  |  |  |  |
| TRGHDRK | Are there any unraised drinking troughs in fields |  |  |  |  |  |  |
|  | No | 158 | 6.61 | 2231 | 93.39 | 2389 | 81.40 |
|  | Yes | 34 | 6.23 | 512 | 93.77 | 546 | 18.60 |
|  |  |  |  |  |  |  |  |
| NATUDRK | Do cattle drink from natural sources of water |  |  |  |  |  |  |
|  | No | 107 | 6.63 | 1507 | 93.37 | 1614 | 54.99 |
|  | Yes | 85 | 6.43 | 1236 | 93.57 | 1321 | 45.01 |
|  |  |  |  |  |  |  |  |
| BVDPOS | Has there ever been an animal on the farm detected as persistently infected with Bovine Viral Diarrhoea (BVD) or Mucosal disease |  |  |  |  |  |  |
|  | No | 153 | 6.36 | 2253 | 93.64 | 2406 | 81.98 |
|  | Yes or don’t know | 39 | 7.37 | 490 | 92.63 | 529 | 18.02 |
|  |  |  |  |  |  |  |  |
| BVDVAC | Is BVD vaccination carried out on the farm |  |  |  |  |  |  |
|  | No | 120 | 5.91 | 1911 | 94.09 | 2031 | 69.20 |
|  | Yes | 72 | 7.96 | 832 | 92.04 | 904 | 30.80 |
|  |  |  |  |  |  |  |  |
| IBRVAC | Is IBR vaccination carried out on the farm |  |  |  |  |  |  |
|  | No | 105 | 5.14 | 1939 | 94.86 | 2044 | 69.64 |
|  | Yes | 87 | 9.76 | 804 | 90.24 | 891 | 30.36 |
|  |  |  |  |  |  |  |  |
| JOHPOS | Has there ever been a case of Johne’s disease in the herd |  |  |  |  |  |  |
|  | No | 162 | 6.00 | 2540 | 94.00 | 2702 | 92.06 |
|  | Yes or don’t know | 30 | 12.88 | 203 | 87.12 | 233 | 7.94 |
|  |  |  |  |  |  |  |  |
| LEPPOS | Has there ever been a case of Leptospirosis detected on the farm |  |  |  |  |  |  |
|  | No | 186 | 6.82 | 2540 | 93.18 | 2726 | 92.88 |
|  | Yes or don’t know | 6 | 2.87 | 203 | 97.13 | 209 | 7.12 |
|  |  |  |  |  |  |  |  |
| LEPVAC | Is Leptospirosis vaccination carried out on the farm |  |  |  |  |  |  |
|  | No | 127 | 5.61 | 2137 | 94.39 | 2264 | 77.14 |
|  | Yes | 65 | 9.69 | 606 | 90.31 | 671 | 22.86 |
|  |  |  |  |  |  |  |  |
| FLKTRT | Is fluke treatment carried out on the farm |  |  |  |  |  |  |
|  | No | 3 | 3.33 | 87 | 96.67 | 90 | 3.07 |
|  | Yes | 189 | 6.64 | 2656 | 93.36 | 2845 | 96.93 |
|  |  |  |  |  |  |  |  |
| FLKDOSEf | How many fluke treatments do cattle receive per year |  |  |  |  |  |  |
|  | 0 | 2 | 3.45 | 56 | 96.55 | 58 | 1.98 |
|  | 1 | 119 | 6.88 | 1611 | 93.12 | 1730 | 58.94 |
|  | >=2 | 71 | 6.19 | 1076 | 93.81 | 1147 | 39.08 |
|  |  |  |  |  |  |  |  |
| SHWINHS | Is there any sharing of winter housing |  |  |  |  |  |  |
|  | No | 189 | 6.53 | 2707 | 93.47 | 2896 | 98.67 |
|  | Yes | 3 | 7.69 | 36 | 92.31 | 39 | 1.33 |
|  |  |  |  |  |  |  |  |
| SHEQUIP | Is there any sharing of equipment with other farms |  |  |  |  |  |  |
|  | No | 190 | 6.61 | 2684 | 93.39 | 2874 | 97.92 |
|  | Yes | 2 | 3.28 | 59 | 96.72 | 61 | 2.08 |
|  |  |  |  |  |  |  |  |
| SHWRKRS | Is there any sharing of farm workers |  |  |  |  |  |  |
|  | No | 191 | 6.64 | 2684 | 93.36 | 2875 | 97.96 |
|  | Yes | 1 | 1.67 | 59 | 98.33 | 60 | 2.04 |
|  |  |  |  |  |  |  |  |
| ONELOC | Are all cattle housed at one location |  |  |  |  |  |  |
|  | No | 60 | 8.97 | 609 | 91.03 | 669 | 22.79 |
|  | Yes | 132 | 5.83 | 2134 | 94.17 | 2266 | 77.21 |
|  |  |  |  |  |  |  |  |
| DIFAIRSP | Are cattle in separate airspaces according to stage of production |  |  |  |  |  |  |
|  | No | 50 | 6.13 | 766 | 93.87 | 816 | 27.80 |
|  | Yes | 142 | 6.70 | 1977 | 93.30 | 2119 | 72.20 |
|  |  |  |  |  |  |  |  |
| BADACFD | Could badgers access cattle feeding area |  |  |  |  |  |  |
|  | No | 50 | 5.88 | 800 | 94.12 | 850 | 28.96 |
|  | Yes | 142 | 6.81 | 1943 | 93.19 | 2085 | 71.04 |
|  |  |  |  |  |  |  |  |
| FEEDGRND | Is the general feeding practise to place feed on the ground |  |  |  |  |  |  |
|  | No | 30 | 5.12 | 556 | 94.88 | 586 | 19.97 |
|  | Yes | 162 | 6.90 | 2187 | 93.10 | 2349 | 80.03 |
|  |  |  |  |  |  |  |  |
| FEEDOUT | Do any sheds have feeding area on the outside of the shed |  |  |  |  |  |  |
|  | No | 98 | 6.01 | 1533 | 93.99 | 1631 | 55.57 |
|  | Yes | 94 | 7.21 | 1210 | 92.79 | 1304 | 44.43 |
|  |  |  |  |  |  |  |  |
| CONENC | Are all concentrate feeds kept in enclosed containers |  |  |  |  |  |  |
|  | No | 15 | 5.24 | 271 | 94.76 | 286 | 9.74 |
|  | Yes | 177 | 6.68 | 2472 | 93.32 | 2649 | 90.26 |
|  |  |  |  |  |  |  |  |
| BADSIL | Could badgers potentially access silage clamps |  |  |  |  |  |  |
|  | No | 29 | 2.94 | 959 | 97.06 | 988 | 33.66 |
|  | Yes | 163 | 8.37 | 1784 | 91.63 | 1947 | 66.34 |
|  |  |  |  |  |  |  |  |
| WILSEC | Are cattle sheds and feed stores secure against wildlife entry |  |  |  |  |  |  |
|  | No | 112 | 6.97 | 1496 | 93.03 | 1608 | 54.79 |
|  | Yes | 80 | 6.03 | 1247 | 93.97 | 1327 | 45.21 |
|  |  |  |  |  |  |  |  |
| MININ | Are mineral blocks or lick buckets used indoors |  |  |  |  |  |  |
|  | No | 148 | 6.95 | 1981 | 93.05 | 2129 | 72.54 |
|  | Yes | 44 | 5.46 | 762 | 94.54 | 806 | 27.46 |
|  |  |  |  |  |  |  |  |
| MINOUT | Are mineral blocks or lick buckets used at pasture |  |  |  |  |  |  |
|  | No | 104 | 6.51 | 1494 | 93.49 | 1598 | 54.45 |
|  | Yes | 88 | 6.58 | 1249 | 93.42 | 1337 | 45.55 |
|  |  |  |  |  |  |  |  |
| SHDSEC | Are cattle sheds secured to prevent badger entry at night |  |  |  |  |  |  |
|  | No | 138 | 6.56 | 1966 | 93.44 | 2104 | 71.69 |
|  | Yes | 54 | 6.50 | 777 | 93.50 | 831 | 28.31 |
|  |  |  |  |  |  |  |  |
| SHDCD | Are sheds washed and disinfected annually |  |  |  |  |  |  |
|  | No | 20 | 5.36 | 353 | 94.64 | 373 | 12.71 |
|  | Yes | 172 | 6.71 | 2390 | 93.29 | 2562 | 87.29 |
|  |  |  |  |  |  |  |  |
| VISCD | Are visitors entering and leaving farm cleansing and disinfecting |  |  |  |  |  |  |
|  | No | 27 | 6.46 | 391 | 93.54 | 418 | 14.24 |
|  | Yes | 165 | 6.56 | 2352 | 93.44 | 2517 | 85.76 |
|  |  |  |  |  |  |  |  |
| SLURCON | Are contractors used to spread slurry or manure |  |  |  |  |  |  |
|  | No | 107 | 6.30 | 1592 | 93.70 | 1699 | 57.89 |
|  | Yes | 85 | 6.88 | 1151 | 93.12 | 1236 | 42.11 |
|  |  |  |  |  |  |  |  |
| SLURGZMT | Is slurry applied to grazing ground. |  |  |  |  |  |  |
|  | No | 22 | 4.01 | 527 | 95.99 | 549 | 18.71 |
|  | Yes | 170 | 7.12 | 2216 | 92.88 | 2386 | 81.29 |
|  |  |  |  |  |  |  |  |
| MANGRZ | Is manure spread on grazing ground |  |  |  |  |  |  |
|  | No or not applicable | 105 | 6.71 | 1459 | 93.29 | 1564 | 53.29 |
|  | Yes | 87 | 6.35 | 1284 | 93.65 | 1371 | 46.71 |
|  |  |  |  |  |  |  |  |
| SLURGRZ | Do cattle ever have access to grazing ground on which fresh slurry is applied? |  |  |  |  |  |  |
|  | No or not applicable | 166 | 6.45 | 2407 | 93.55 | 2573 | 87.67 |
|  | Yes | 26 | 7.18 | 336 | 92.82 | 362 | 12.33 |
|  |  |  |  |  |  |  |  |
| SLURTIME | Is slurry applied in spring time as opposed to continuously over year? |  |  |  |  |  |  |
|  | No or not applicable | 54 | 7.64 | 653 | 92.36 | 707 | 24.09 |
|  | Yes | 138 | 6.19 | 2090 | 93.81 | 2228 | 75.91 |
|  |  |  |  |  |  |  |  |
| SLUROTH | Is slurry or manure applied that is produced on other farms |  |  |  |  |  |  |
|  | No | 185 | 6.57 | 2632 | 93.43 | 2817 | 95.98 |
|  | Yes | 7 | 5.93 | 111 | 94.07 | 118 | 4.02 |
|  |  |  |  |  |  |  |  |
| PURBULL | Has a bull been purchased in the past 5 years |  |  |  |  |  |  |
|  | No | 60 | 5.05 | 1128 | 94.95 | 1188 | 40.48 |
|  | Yes | 132 | 7.56 | 1615 | 92.44 | 1747 | 59.52 |
|  |  |  |  |  |  |  |  |
| HIRBULL | Has a bull been purchased or hired during last 5 years |  |  |  |  |  |  |
|  | No | 190 | 6.55 | 2710 | 93.45 | 2900 | 98.81 |
|  | Yes | 2 | 5.71 | 33 | 94.29 | 35 | 1.19 |
|  |  |  |  |  |  |  |  |
| PURCOW | Have replacement breeding cows or heifers been purchased in last 5 years |  |  |  |  |  |  |
|  | No | 101 | 6.76 | 1394 | 93.24 | 1495 | 50.94 |
|  | Yes | 91 | 6.32 | 1349 | 93.68 | 1440 | 49.06 |
|  |  |  |  |  |  |  |  |
| PURCALF | Have calves been purchased in the previous 5 years |  |  |  |  |  |  |
|  | No | 131 | 6.70 | 1825 | 93.30 | 1956 | 66.64 |
|  | Yes | 61 | 6.23 | 918 | 93.77 | 979 | 33.36 |
|  |  |  |  |  |  |  |  |
| REARCALF | Is the purchase and rearing of calves part of the farm business |  |  |  |  |  |  |
|  | No | 163 | 6.49 | 2348 | 93.51 | 2511 | 85.55 |
|  | Yes | 29 | 6.84 | 395 | 93.16 | 424 | 14.45 |
|  |  |  |  |  |  |  |  |
| PURSTORE | Have store cattle (cattle for fattening) been purchased in the previous 5 years |  |  |  |  |  |  |
|  | No | 127 | 7.40 | 1589 | 92.60 | 1716 | 58.47 |
|  | Yes | 65 | 5.33 | 1154 | 94.67 | 1219 | 41.53 |
|  |  |  |  |  |  |  |  |
| PURMART | Has any purchase been made from a cattle market in the previous 5 years |  |  |  |  |  |  |
|  | No | 54 | 6.55 | 770 | 93.45 | 824 | 28.07 |
|  | Yes | 138 | 6.54 | 1973 | 93.46 | 2111 | 71.93 |
|  |  |  |  |  |  |  |  |
| PURDIR | Has there been any purchases directly from another herd and was an enquiry made if that herd had a history of TB breakdowns |  |  |  |  |  |  |
|  | No purchase | 49 | 6.13 | 750 | 93.87 | 799 | 27.22 |
|  | Yes but no enquiry | 69 | 5.97 | 1086 | 94.03 | 1155 | 39.35 |
|  | Yes plus enquiry | 74 | 7.54 | 907 | 92.46 | 981 | 33.42 |
|  |  |  |  |  |  |  |  |
| PURDEAL | Have cattle been purchased from a dealer in the previous 5 years |  |  |  |  |  |  |
|  | No | 167 | 6.42 | 2436 | 93.58 | 2603 | 88.69 |
|  | Yes | 25 | 7.53 | 307 | 92.47 | 332 | 11.31 |
|  |  |  |  |  |  |  |  |
| ISOPOS | Are animals isolated post purchase |  |  |  |  |  |  |
|  | No | 66 | 6.26 | 988 | 93.74 | 1054 | 35.91 |
|  | Yes | 113 | 6.69 | 1576 | 93.31 | 1689 | 57.55 |
|  | Not Applicable | 13 | 6.77 | 179 | 93.23 | 192 | 6.54 |
|  |  |  |  |  |  |  |  |
| TOTPARf | Total number of land parcels on which animals are grazed per year |  |  |  |  |  |  |
|  | 1 | 33 | 4.09 | 774 | 95.91 | 807 | 27.50 |
|  | 2 | 45 | 6.12 | 690 | 93.88 | 735 | 25.04 |
|  | 3 | 57 | 9.06 | 572 | 90.94 | 629 | 21.43 |
|  | >=4 | 57 | 7.46 | 707 | 92.54 | 764 | 26.03 |
|  |  |  |  |  |  |  |  |
| RENTGRND | Is extra ground rented and is it >5km from main farm |  |  |  |  |  |  |
|  | None rented | 82 | 5.13 | 1518 | 94.88 | 1600 | 54.51 |
|  | Yes and >5km | 43 | 7.83 | 506 | 92.17 | 549 | 18.71 |
|  | Yes and <5km | 67 | 8.52 | 719 | 91.48 | 786 | 26.78 |
|  |  |  |  |  |  |  |  |
| TBGRND | Is there particular parcels of ground that are associated with cattle becoming infected and have these been put out of use |  |  |  |  |  |  |
|  | None associated | 139 | 5.72 | 2293 | 94.28 | 2432 | 82.86 |
|  | Yes and used | 53 | 10.54 | 450 | 89.46 | 503 | 17.14 |
|  |  |  |  |  |  |  |  |
| FENCEUP | Have any fences with a neighbour been upgraded in the last 3 years and did this upgrade involve installation of a complete new fence |  |  |  |  |  |  |
|  | No | 31 | 4.30 | 690 | 95.70 | 721 | 24.57 |
|  | Yes plus full upgrade of fences | 77 | 7.27 | 982 | 92.73 | 1059 | 36.08 |
|  | Yes plus some upgrading of fences | 84 | 7.27 | 1071 | 92.73 | 1155 | 39.35 |
|  |  |  |  |  |  |  |  |
| FENCEDB | Are there any double fences around the farm perimeter boundary |  |  |  |  |  |  |
|  | No | 108 | 6.07 | 1672 | 93.93 | 1780 | 60.65 |
|  | Yes | 84 | 7.27 | 1071 | 92.73 | 1155 | 39.35 |
|  |  |  |  |  |  |  |  |
| CATCON | Were there any break ins or break outs of cattle to from a neighbour in the previous grazing season |  |  |  |  |  |  |
|  | No | 169 | 6.41 | 2467 | 93.59 | 2636 | 89.81 |
|  | Yes | 23 | 7.69 | 276 | 92.31 | 299 | 10.19 |
|  |  |  |  |  |  |  |  |
| NOSECON | Can cattle have nose to nose contact with others during grazing season and is this possible with > 3 herds |  |  |  |  |  |  |
|  | No | 30 | 5.35 | 531 | 94.65 | 561 | 19.11 |
|  | Yes and <=3 | 46 | 5.13 | 851 | 94.87 | 897 | 30.56 |
|  | Yes and >3 | 116 | 7.85 | 1361 | 92.15 | 1477 | 50.32 |
|  |  |  |  |  |  |  |  |
| SILCUTS | Number of silage cuts taken per year |  |  |  |  |  |  |
|  | <=2 | 141 | 5.88 | 2259 | 94.13 | 2400 | 81.77 |
|  | >2 | 51 | 9.53 | 484 | 90.47 | 535 | 18.23 |
|  |  |  |  |  |  |  |  |
| SILGRZ | Are silage fields after grazed with cattle |  |  |  |  |  |  |
|  | No or not applicable | 15 | 3.94 | 366 | 96.06 | 381 | 12.98 |
|  | Yes | 177 | 6.93 | 2377 | 93.07 | 2554 | 87.02 |
|  |  |  |  |  |  |  |  |
| BADSETT | Are there any badger setts on the farm |  |  |  |  |  |  |
|  | No | 112 | 5.45 | 1942 | 94.55 | 2054 | 69.98 |
|  | Yes | 80 | 9.08 | 801 | 90.92 | 881 | 30.02 |
|  |  |  |  |  |  |  |  |
|  |  |  |  |  |  |  |  |
| BADLOCAL | Is there badger setts in the locality that are not on this farm |  |  |  |  |  |  |
|  | No | 110 | 5.51 | 1887 | 94.49 | 1997 | 68.04 |
|  | Yes | 82 | 8.74 | 856 | 91.26 | 938 | 31.96 |
|  |  |  |  |  |  |  |  |
| BADLIVON | Has there been a live badger seen on the farm in the past 3 years |  |  |  |  |  |  |
|  | No | 129 | 5.69 | 2137 | 94.31 | 2266 | 77.21 |
|  | Yes | 63 | 9.42 | 606 | 90.58 | 669 | 22.79 |
|  |  |  |  |  |  |  |  |
| BADLIVOT | Has there been a live badger seen on a neighbouring farms in the past 3 years |  |  |  |  |  |  |
|  | No | 161 | 6.20 | 2435 | 93.80 | 2596 | 88.45 |
|  | Yes | 31 | 9.14 | 308 | 90.86 | 339 | 11.55 |
|  |  |  |  |  |  |  |  |
| BADDEAD | Has there been a dead badger on the farm in the last 3 years |  |  |  |  |  |  |
|  | No | 162 | 5.86 | 2602 | 94.14 | 2764 | 94.17 |
|  | Yes | 30 | 17.54 | 141 | 82.46 | 171 | 5.83 |
|  |  |  |  |  |  |  |  |
| BADROAD | Has there been a dead badger on a road within a mile of the farm in the past 3 years |  |  |  |  |  |  |
|  | No | 59 | 5.03 | 1113 | 94.97 | 1172 | 39.93 |
|  | Yes | 133 | 7.54 | 1630 | 92.46 | 1763 | 60.07 |
|  |  |  |  |  |  |  |  |
| BADHOU | Has there been a badger seen in any of the sheds in the last 3 years |  |  |  |  |  |  |
|  | No | 186 | 6.41 | 2715 | 93.59 | 2901 | 98.84 |
|  | Yes | 6 | 17.65 | 28 | 82.35 | 34 | 1.16 |
|  |  |  |  |  |  |  |  |
| BADSTOR | Has there been a badger seen in the feed store in the previous 3 years |  |  |  |  |  |  |
|  | No or not applicable | 192 | 6.55 | 2740 | 93.45 | 2932 | 99.90 |
|  | Yes | 0 | 0.00 | 3 | ##### | 3 | 0.10 |
|  |  |  |  |  |  |  |  |
| SETTFEN | Are badger setts fenced off |  |  |  |  |  |  |
|  | No or not applicable | 185 | 6.49 | 2664 | 93.51 | 2849 | 97.07 |
|  | Yes | 7 | 8.14 | 79 | 91.86 | 86 | 2.93 |
|  |  |  |  |  |  |  |  |
| LATFEN | Are badger latrines fenced off |  |  |  |  |  |  |
|  | No or not applicable | 190 | 6.58 | 2698 | 93.42 | 2888 | 98.40 |
|  | Yes | 2 | 4.26 | 45 | 95.74 | 47 | 1.60 |
|  |  |  |  |  |  |  |  |
| DEERPRES | Have there been any deer seen on the farm in the past 3 years |  |  |  |  |  |  |
|  | No | 146 | 6.37 | 2145 | 93.63 | 2291 | 78.06 |
|  | Yes | 46 | 7.14 | 598 | 92.86 | 644 | 21.94 |
|  |  |  |  |  |  |  |  |
| WOODPRS | Is there any woodland on the farm or within a mile of the farm |  |  |  |  |  |  |
|  | No | 95 | 7.61 | 1154 | 92.39 | 1249 | 42.56 |
|  | Yes | 97 | 5.75 | 1589 | 94.25 | 1686 | 57.44 |
|  |  |  |  |  |  |  |  |
| DVOHRD | District Veterinary Office of the breakdown herd |  |  |  |  |  |  |
|  | 01, 08, 09 | 37 | 4.47 | 790 | 95.53 | 827 | 28.18 |
|  | 04, 05, 07, 10 | 88 | 6.26 | 1317 | 93.74 | 1405 | 47.87 |
|  | 02, 03,06 | 67 | 9.53 | 636 | 90.47 | 703 | 23.95 |
|  |  |  |  |  |  |  |  |
| Num1RiskSource | First choice as source of infection by investigating Veterinary Officer |  |  |  |  |  |  |
|  | Source of infection is not established or Other(including Deer source) | 44 | 4.88 | 857 | 95.12 | 901 | 30.70 |
|  | Source of infection cattle to cattle spread | 39 | 5.86 | 627 | 94.14 | 666 | 22.69 |
|  | Source of infection purchase of infected animal(s) | 33 | 6.35 | 487 | 93.65 | 520 | 17.72 |
|  | Source of infection carryover of previous infection | 40 | 18.78 | 173 | 81.22 | 213 | 7.26 |
|  | Source of infection is badgers | 36 | 5.67 | 599 | 94.33 | 635 | 21.64 |
|  |  |  |  |  |  |  |  |
| Num2RiskSource | Second choice as source of infection by investigating Veterinary Officer |  |  |  |  |  |  |
|  | Source of infection is not established or Other(including Deer source) | 12 | 4.00 | 288 | 96.00 | 300 | 10.22 |
|  | Source of infection cattle to cattle spread | 45 | 8.46 | 487 | 91.54 | 532 | 18.13 |
|  | Source of infection purchase of infected animal(s) | 5 | 3.09 | 157 | 96.91 | 162 | 5.52 |
|  | Source of infection carryover of previous infection | 25 | 18.52 | 110 | 81.48 | 135 | 4.60 |
|  | Source of infection is badgers | 44 | 6.75 | 608 | 93.25 | 652 | 22.21 |
|  | No second choice source selected | 61 | 5.29 | 1093 | 94.71 | 1154 | 39.32 |
|  |  |  |  |  |  |  |  |

**Supplementary Table S2:** Table showing on farm questionnaire used for on farm data collection.

TB Disease Investigation For

| Name: ____________________________________________  Herd Number: ______________________________________  Address: ___________________________________________  Associated Herd Number(s): ___________________________  Date OTS/OTW applied: _____________________  Map Reference (centre of farm activity in the form **A 123 456**): _____________________  DVO: Patch (Number followed by capital letter): _______________  Herd keeper contact telephone number(s): _________________________________________  H&S status on herd: Y N. Details ______________________________________________ |
| --- |
| **Verification of VO phone call to breakdown herd keeper and of passing of form to SAHWI.**  All points as detailed in Staff Instruction communicated to herd keeper 🗆  VO Signature ­­­­­­­­­­­­­­­­­­­­­­­­­­­­­­­­_______________________. Aphis User code ______. Date _________  Date passed to SAHWI: _______________ |
| **Breakdown History**  Date of last TB breakdown. From_________; to ­­­­___________  Strain types isolated during previous breakdown: ­­­­­­­­­­­­­­­­­­­­­­­__________________________  Most likely source of infection at previous breakdown: _____________________  Total number of reactors/LRSs at previous breakdown: _______________  At risk to how many current TB breakdowns ___________  Have reactors been RI in previous tests? ____________________ |

| **AHWI APHIS checks:**  At what date did breakdown start: ___________________  Herd size and herd type: _____________________________  Did this breakdown initiate with a skin test or an LRS?   1. Test date: _________ Test type: __________ Number of reactors: _____________   or   1. LRS ____________________________   Reactor(s): Homebred Purchased  What is the Test ID of the test initiating this incident? ________________  Date and type of previous herd test: _________________  List any other farmed species kept by breakdown herd keeper. ­­­­­­­­­­­­­­­­­­­_____________________________  Movement of livestock onto farm in last 5 years. Y N   1. Source: Herds Market Dealer Import 2. Frequency: Weekly Monthly Quarterly Annually 3. Comment:   Movement of livestock off the farm. Y N   1. Destination: Farm Market Abattoir Export Dealer Show   **AHWI checklist: Summary of instructions.**   1. Update the attached maps 🗆 2. Last housing date ______________ 🗆 3. Ensure all land parcels have been mapped, named & acreage noted 🗆 4. Note usage of each land parcel of breakdown and neighbouring herds 🗆   **S**  **R**   1. Mark reactors on map with and badger setts with 🗆 2. Confirm who is using adjacent land parcels 🗆 3. Note boundary types for entire perimeter of breakdown 🗆 4. Fill in the investigation form 🗆 5. Update list of contiguous herds on Appendix 1 🗆 6. Return completed forms to Patch VO Date ________________ 🗆 7. Notify SAHWI of handover date 🗆 |
| --- |

**DISEASE INVESTIGATION QUESTIONNAIRE**

| **Section A. General management practices** | | | | | | | | | |
| --- | --- | --- | --- | --- | --- | --- | --- | --- | --- |
|  | 1. What farming activities do you practice? 2. Do you have registered pedigree animals? | Milk  Y | Suckler cows  N | | Beef fattening | | | Other  If yes give %: | |
|  | Herd size over the past 10 years? | Increased | Same | | Decreased | | | | |
|  | Are any cattle wintered outside? | Most | Some | | None | | | | |
|  | Is part of your herd managed as a separate unit at an outfarm, for example beef animals managed separately from the milking herd? | Y | N | | Details: | | | | |
|  | Has TB affected one sub-group of your herd?  (Sub-group here means production groups of cattle e.g. milking cows, dry cows, maiden heifers, beef bullocks etc) | Y | N | | Elaborate on batchings: | | | | |
|  | If TB has affected a sub-group, which age group is this? | Adult cattle: Cows + Bulls | Cattle 15 to 30 months | | Cattle less than 15 months | | | | N/A |
|  | 1. Which grazing patterns do you practice?   b. Do you use zero grazing for any of your cattle groups? | Rotational  Y | Set Stocking  N | | Strip Grazing | | N/A  Comment: | | |
|  | Do you operate any mixed grazing of cattle and sheep? | Y | N | |  | | | | |
|  | 1. Do you ever feed meal to cattle on the ground or in unraised feeding troughs at pasture? 2. Do you use calf creep feeders at pasture? | Y  Y | N  N | |  | | | | |
|  | If feeding meal to cattle on the ground at pasture which group of stock does it apply to? | Adult cattle: Cows + Bulls | Cattle less than 30 months | | All age groups of cattle | | | N/A | |
|  | 1. Do you have any unraised drinking troughs in your fields? 2. Do cattle drink from natural sources of water? | Y  Y | N  N | |  | | | | |
|  | 1. Have you ever had an animal detected as persistently infected with BVD (Bovine Viral Diarrhoea), or a case of Mucosal Disease in your herd)   b. Do you vaccinate herd for BVD? | Y  Y | N  N | | Don’t know | | |  | |
|  | Do you vaccinate herd for IBR? | Y | N | |  | | |  | |
|  | Have you ever had a case of Johne’s disease in your herd? | Y | N | | Don’t know | | |  | |
|  | 1. Have you ever had a case of Leptospirosis in your herd?   b. Do you vaccinate herd for Leptospirosis? | Y  Y | N  N | | Don’t know | | |  | |
|  | Do you use fluke treatments on cattle on your farm? | Y | N | |  | | | | |
|  | On a yearly basis how often do you treat your cattle for fluke? | 0 | | 1 | | >=2 | | | |
|  | Do you share:   1. Winter housing 2. Equipment 3. Farm workers | Y  Y  Y | N  N  N | |  | | | | |
| **Section B. The farm yard and housing** | | | | | | | | | |
|  | Do you house all your cattle at one location? | Y | N | | If no, name the locations and list type of stock kept there: | | | | |
|  | Are cattle at all farm locations separated into different sheds/ airspaces according to their age/ stage of production?  **Also note the sheds where the reactors were housed.** | Y | N | | If yes, list which groups share common air spaces: | | | | |
|  | Could badgers access the cattle feeding area? | Y | N | |  | | | | |
|  | Is your general feeding practice in most sheds to feed on the ground? | Y | N | |  | | | | |
|  | Do you have any sheds where the feeding area is on the outside of the shed and could be accessed by wildlife? | Y | N | |  | | | | |
|  | Do you keep all concentrate feed in enclosed containers? | Y | N | |  | | | | |
|  | Could badgers potentially access silage clamps? | Y | N | |  | | | | |
|  | Are cattle sheds and feed stores capable of being secured so that they do not allow wildlife entry? | Y | N | |  | | | | |
|  | a. Are mineral blocks/ lick buckets used indoors?  b. Are mineral blocks/ lick buckets used at pasture? | Y  Y | N  N | |  | | | | |
|  | Are cattle sheds secured at night with adequate doors or electric fences so as to prevent entry of badgers? | Y | N | |  | | | | |
|  | Are livestock sheds washed and disinfected annually? | Y | N | |  | | | | |
|  | Are all visitors coming onto the farm cleaning and disinfecting every time they enter or leave the farm? | Y | N | |  | | | | |
| **Section C. On farm slurry/manure production** | | | | | | | | | |
|  | Are contractors used to spread slurry/manure? | Y | N | |  | | | | |
|  | 1. Is slurry applied to grazing ground? 2. If so, which methods are used? N/A 3. Is manure spread on grazing ground? | Y  Injector  Y | N  Trailing shoe  N | | Inverted splash-plate  N/A | | | Umbilical system | |
|  | Do cattle ever have access to grazing ground on which fresh slurry is applied? | Y | N | | N/A | | | | |
|  | Is slurry/manure applied mostly in spring time rather than continuously over the grazing season? | Y | N | | N/A | | | | |
|  | Do you use slurry/manure that is produced on other farms? | Y | N | | If yes, what is the source? | | | | |
| **Section D. Movement of cattle** | | | | | | | | | |
|  | Have you purchased a bull(s) in the last 5 years? | Y | N | |  | | | | |
|  | Have you ever hired or borrowed a bull during the breeding season in the last 5 years? | Y | N | | If yes, please state source: | | | | |
|  | Have you purchased replacement breeding cows or heifers in the last 5 years? | Y | N | |  | | | | |
|  | Have you purchased calves in the last 5 years? | Y | N | |  | | | | |
|  | Is the purchase and rearing of drop calves part of your business? | Y | N | |  | | | | |
|  | Have you purchased stores in the last 5 years? | Y | N | |  | | | | |
|  | Have you purchased directly from a mart in the last 5 years? | Y | N | |  | | | | |
|  | 1. Have you purchased directly from another herd in the last 5 years? 2. If yes, did you enquire about the TB status of that herd? | Y  Y  Detail practice implemented: | N  N | |  | | | | |
|  | Have you purchased cattle directly from a dealer in the last 5 years? | Y | N | | Clarification: This does not include using a dealer as an agent to purchase animals on the herd keepers behalf | | | | |
|  | Do you isolate animals post purchase? | Y | N | | N/A | | | | |
| **Section E. Contact with other cattle** | | | | | | | | | |
|  | What is the total number of land parcels on which your animals were grazed in the last grazing season? | 1 | 2 | | 3 | | | >=4 | |
|  | 1. Do you rent any extra grazing ground? 2. If you rent extra grazing ground is any parcel more than 3 miles from your home farm unit? | Y  Y | N  N | |  | | |  | |
|  | Do you have particular parcels of land you associate with TB in your cattle?  If you answered YES have you stopped using this land for cattle? | Y  Y | N  N | |  | | |  | |
|  | Have you upgraded any boundary fences between yourself and a neighbouring herd in the last 3 years? | Y | N | |  | | | | |
|  | If you have upgraded boundary fences in the last 3 years did this upgrade involve installation of a completely new fence between yourself and a neighbouring herd? | Y | N | |  | | |  | |
|  | Do you have double fences along your perimeter boundary? | Y | N | |  | | | | |
|  | Did you have any cattle break-ins or break-outs during the current or previous grazing season? | Y | N | |  | | | | |
|  | 1. Can your cattle have nose to nose contact with neighbouring herds during the grazing season? 2. If yes, is contact possible with more than 3 herds? | Y  Y | N  N | |  | | |  | |
|  | 1. How many cuts of silage do you take? 2. Do you after-graze your silage fields with cattle? | 0 1 2 3 4  Y N N/A | | | | | | | |
| **Section F. Wildlife** | | | | | | | | | |
|  | Are you aware of any badger setts on your farm? | Y | N | |  | | | | |
|  | Are you aware of any badger setts in the locality but not on your farm? | Y | N | |  | | | | |
|  | Have you seen a live badger on your farm in the last 3 years? | Y | N | |  | | | | |
|  | Have you seen a live badger on neighbouring farms in the last 3 years? | Y | N | |  | | | | |
|  | Have you seen a dead badger on your farm in the last 3 years? | Y | N | |  | | | | |
|  | Have you seen any dead badgers seen on a public road within a mile of any of your land in the last 3 years? | Y | N | |  | | | | |
|  | Have you seen any badgers in any of your cattle houses during the housing period in the last 3 years? | Y | N | |  | | | | |
|  | Have you seen a badger in your feed store in the last 3 years? | Y | N | | N/A – no feed store | | | | |
|  | 1. Do you fence off badger setts? 2. Do you fence off badger latrines? | Y  Y | N  N | | N/A  N/A | | | | |
|  | Have you noticed any deer on your farmland in the last 3 years? | Y | N | |  | | | | |
|  | Have you any woodland on your farm or within a mile of your farm? | Y | N | |  | | | | |
| AHWI Comments | | | | | | | | | |
| Herd keeper informed of future VO visit.  (S)AHWI signature _____________________. APHIS user code ­­­­­­­­­­­__________. Date __________. | | | | | | | | | |
| **Section G. VO Assessment** | | | | | | | | | |
|  | Score the general cleanliness of farm yard(s) 1-3. (3 being the most clean) |  | | | | | | | |
|  | Is the herd managed as separate units? | Y | N | |  | | | | |
|  | Did the TB outbreak on this farm affect mainly one particular subgroup of animals on the farm? | Y | N | |  | | | | |
|  | Has the farmer implemented any biosecurity measures on the farm?   1. C&D points on farm 2. Badgerproofing farmyard 3. Badgerproofing feedstore 4. Fencing off badger setts 5. Fencing off latrines 6. Minimising movement between land parcels 7. Minimising purchase of cattle 8. Biosecure farm boundaries 9. Minimising sharing of machinery/facilities/staff | Y  Y  Y  Y  Y  Y  Y  Y  Y | N  N  N  N  N  N    N  N  N | | Comments:  N/A  N/A  N/A  N/A | | | | |
|  | Based on all available information in relation to this breakdown, what is the most likely source of infection?  Rank the most likely source as 1 continuing to 2,3,4 etc, for any other sources you consider possible but less likely.  If you consider a source unlikely do not give it any rank. | 1. Cattle to cattle spread 2. Purchase of infected animal(s) 3. Carryover of previous infection 4. Badgers 5. Deer 6. Not established 7. Other: ______________________________________________________ | | |  | | | | |
| VO check that form is fully completed and accurate  VO signature _____________________. APHIS user code ­­­­­­­­­­­__________. Date __________. | | | | | | | | | |

| **Further Breakdown Details – breakdown summary:**  Herd now OTF / OTS / OTW (Circle one)  PM results:  Histology results:  Bacteriology results:  Total no. of reactors taken:  Total number of LRS:  **Summary of Relevant Items from Investigation Form:**  **Advice given to herd keeper at VO visit:** |
| --- |

| **Section H. CSB section** | |
| --- | --- |
| Updated mapping contig list attached | Yes No |
| Scanned to HPRM  Document number  Date e-mailed to HQ | Yes No  _______________________  _______________________ |
| CSB signature _____________________. APHIS user code ­­­­­­­­­­­__________. Date __________. | |

**Supplementary S3:** Model Diagnostics: Residual plot

A binned residual plot was produced as part of model diagnostics. This plot shown below shows the grouped residuals, with the line representing 2 standard deviations from zero residual.


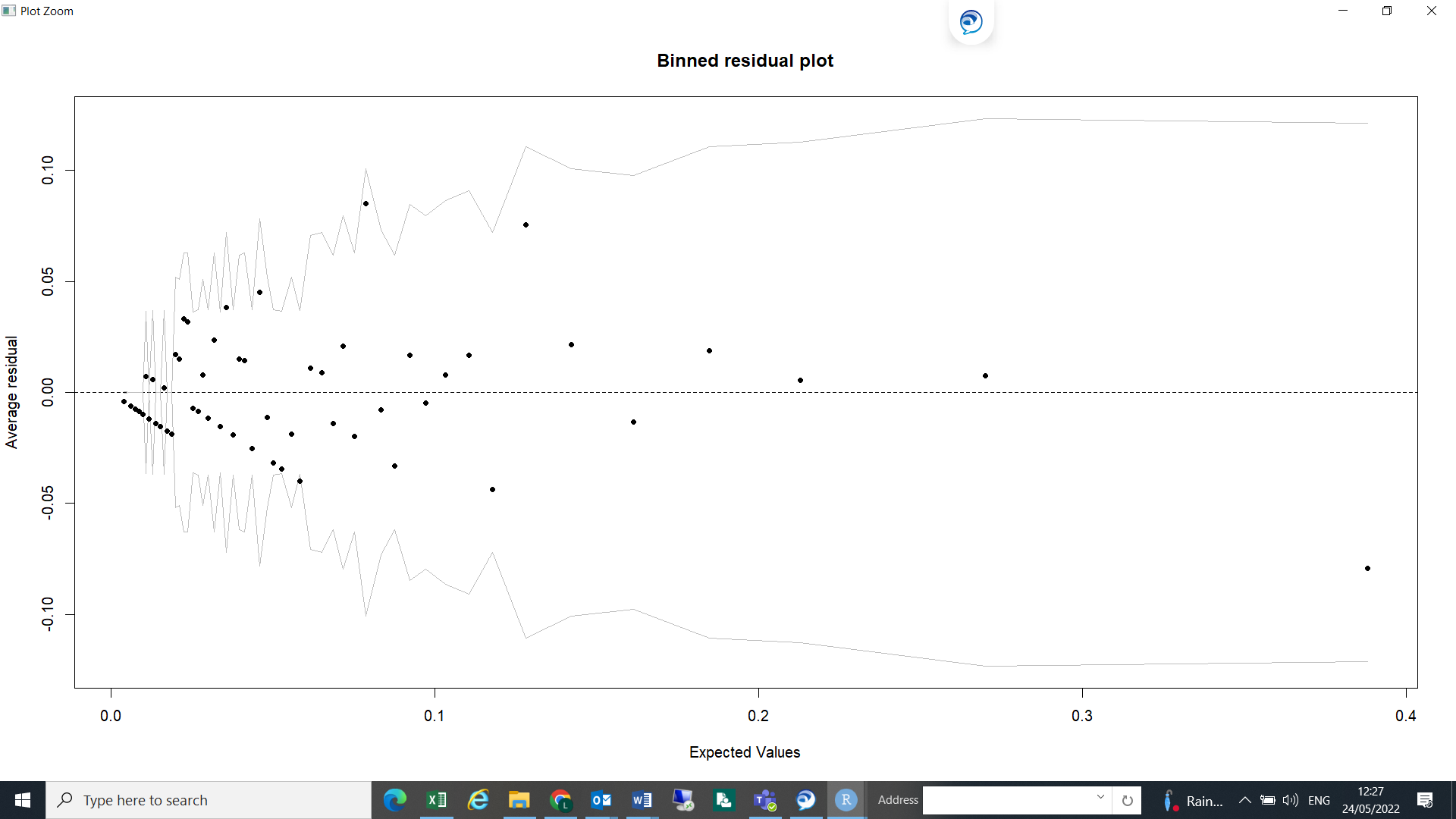


Reference: “In logistic regression, as with linear regression, the residuals can be defined as observed minus expected values. The data are discrete and so are the residuals. As a result, plots of raw residuals from logistic regression are generally not useful. The binned residuals plot instead, after dividing the data into categories (bins) based on their fitted values, the average residual versus the average fitted value for each bin.” *Ref: Binned residual plots are also discussed on page 97 in Gelman and Hill.*

**Supplementary S4:** Review comment and reply relating to number of significant variables

The reviewer pointed out that authors correctly assert having a large number of significant variables increases the chances of a type 1 error. Could there have been a method applied for adjusting for this (e.g a Bonferroni correction). Is there any reason why such adjustments weren't made?

The authors pointed out that the main objective of this point was simply to raise the reader’s awareness that in a study of this nature, with 13 variables and a 5% cut-off, there is always a risk that one or more could be in there simply by chance. We felt this an important point to raise as it reminds the reader to more critically evaluate the results.

In a personal communication from our statistician for this work (AGordon) he felt was that using a correction technique would not be of a lot of benefit in the case of logistic regression (he says he has only ever used Bonferroni in multiple comparison tests after an ANOVA, never in regression work) and that the general premise we are trying to communicate would still be relevant anyway.
